# Engaging Terminally Ill Patients in End of Life Talk: How Experienced Palliative Medicine Doctors Navigate the Dilemma of Promoting Discussions about Dying

**DOI:** 10.1371/journal.pone.0156174

**Published:** 2016-05-31

**Authors:** Marco Pino, Ruth Parry, Victoria Land, Christina Faull, Luke Feathers, Jane Seymour

**Affiliations:** 1 Department of Social Sciences, Loughborough University, Loughborough, United Kingdom; 2 School of Health Sciences, University of Nottingham, Nottingham, United Kingdom; 3 LOROS hospice, Leicester, United Kingdom; 4 School of Nursing and Midwifery, The University of Sheffield, Sheffield, United Kingdom; University of British Columbia, CANADA

## Abstract

**Objective:**

To examine how palliative medicine doctors engage patients in end-of-life (hereon, EoL) talk. To examine whether the practice of “eliciting and responding to cues”, which has been widely advocated in the EoL care literature, promotes EoL talk.

**Design:**

Conversation analysis of video- and audio-recorded consultations.

**Participants:**

Unselected terminally ill patients and their companions in consultation with experienced palliative medicine doctors.

**Setting:**

Outpatient clinic, day therapy clinic, and inpatient unit of a single English hospice.

**Results:**

Doctors most commonly promoted EoL talk through *open elaboration solicitations*; these created opportunities for patients to introduce–then later further articulate–EoL considerations in such a way that doctors did not overtly ask about EoL matters. Importantly, the wording of elaboration solicitations avoided assuming that patients had EoL concerns. If a patient responded to open elaboration solicitations without introducing EoL considerations, doctors sometimes pursued EoL talk by switching to a less participatory and more presumptive type of solicitation, which suggested the patient might have EoL concerns. These more overt solicitations were used only later in consultations, which indicates that doctors give precedence to patients volunteering EoL considerations, and offer them opportunities to take the lead in initiating EoL talk. There is evidence that doctors treat elaboration of patients’ talk as a resource for engaging them in EoL conversations. However, there are limitations associated with labelling that talk as “cues” as is common in EoL communication contexts. We examine these limitations and propose “possible EoL considerations” as a descriptively more accurate term.

**Conclusions:**

Through communicating–via open elaboration solicitations–in ways that create opportunities for patients to volunteer EoL considerations, doctors navigate a core dilemma in promoting EoL talk: giving patients opportunities to choose whether to engage in conversations about EoL whilst being sensitive to their communication needs, preferences and state of readiness for such dialogue.

## Introduction

There is consensus that terminally ill patients should be given opportunities to discuss their prognosis and preferences for end-of-life (EoL) care before illness progression reduces their ability to engage in meaningful conversations. [1–3] Guidelines recommend that health care professionals (HCPs) enable patients to discuss feelings, expectations, and care preferences for the EoL. [4–6] Many families have regrets after the patient has died that such opportunities for hearing the patient’s wishes and for making choices and preparations were missed and that professionals had not told them that death could happen soon. [7] However, HCPs may be reluctant to have such conversations, not least because they could be seen as taking away hope or causing emotional harm. [8] This can lead HCPs to wait for patients to introduce EoL matters into the conversation. However, patients vary in their EoL awareness and in their ability, willingness, and readiness to discuss EoL matters; therefore, patients may not always take the initiative of broaching the topic of dying. [9–13] In this debate, HCPs can be caught in a tension between the expectation that EoL talk is actively promoted, and the expectation that this is done in a way that is sensitive to patients’ communication needs and preferences. These considerations lead to a core dilemma in EoL communication. If death is a difficult topic, and patients are often reluctant to raise it, then HCPs should actively invite such discussion; however, precisely because death is a troublesome topic, invitations to discuss it can be inappropriate or very distressing for some patients. [14–16] The study reported here identifies ways that some palliative medicine doctors navigate this dilemma.

Existing guidance and prior research have suggested that HCPs can promote EoL conversations by responding to patients’ cues, the assumption being that the dilemma of initiating EoL talk can be solved by following up on and encouraging the elaboration of perceived hints that patients themselves introduce into the conversation; in this way, talk about death could emerge as an elaboration of something that patients have alluded to, with no need for HCPs to unilaterally introduce the topic. [8, 17–21] This leads us to ask what constitutes a “patient cue”, and whether encouraging the elaboration of cues does indeed lead to promoting EoL talk. There has been substantial work to reach consensus amongst researchers on how to recognise and code cues. [22, 23] As a result, patient cues have been defined as “verbal or nonverbal hints, which suggest an underlying unpleasant emotion and that lack clarity”. [24, 25] There has been no investigation, however, of whether HCPs themselves (i.e. not researchers doing post hoc analysis) observably treat parts of their patients’ talk as cues relevant to EoL concerns in the course of a consultation, and of whether they do indeed use such cues as resources for engaging patients in EoL talk. Does HCPs’ in-consultation communication reveal whether they recognise and utilise aspects of patients’ communication as cues relevant to EoL concerns? To address this question, we analysed recorded interactions between experienced palliative medicine doctors, terminally ill patients, and their companions. We examined how doctors engage patients in EoL talk (defined as talk that participants observably treat as focusing on the patient’s prospect of deterioration and death); whether doctors observably treat parts of their patients’ talk as cues relevant to EoL concerns; and whether soliciting the elaboration of cues is a way of navigating the dilemma of EoL talk initiation (by promoting EoL talk without explicitly inviting it). In this paper we use the term “soliciting” for actions that promote the production of a certain action–specifically we focus on elaboration solicitations, which promote further talk on something that the patient (or a companion) has previously raised. We used conversation analysis (CA), which allows systematic examination of the structure and functions of communication practices. This approach is becoming the gold standard for rigorous study of communication in healthcare. [26–29] CA findings have provided the underpinning evidence for interventions that have proved effective in improving healthcare communication. [30, 31]

## Materials and Methods

### Participants and methods

Ethical approval was obtained from NRES Committee West Midlands—Coventry & Warwickshire, UK (ref 14/WM/0128). Patients having an inpatient, outpatient, or day therapy consultation with a consultant in one English hospice were invited to participate if they had capacity to consent, were able to speak and understand English, and were judged by the care team not to be in acute distress. All patients had a terminal diagnosis and had been referred to the hospice by a GP, hospital doctor or nurse specialist, in order to review difficult symptoms or for help with planning future care. We employed a retrospective consent procedure, which was approved by the ethics committee, because it was not always possible to contact patients before the day of their appointment. [32] In this procedure, a researcher provided information about the study and sought verbal assent from each patient and their accompanying family members or friends (companions henceforth) to record their conversation with the doctor. If all parties agreed, their conversation with the doctor was recorded that day. The researcher also gave the patient (and companions when present) written information about the study. They were then given time to consider whether they were happy for the research team to retain and use the recording. At least one day after recording, the researcher contacted the patient (and companions) to make an appointment where written informed consent would be sought. At this appointment, the researcher informed the patient and any companion about the intended uses of the recordings. Informed consent forms allowed them to separately authorise different levels of use (e.g. data analysis, use of clips of the recordings for dissemination of findings and within communication skills training resources). We used both a prospective and retrospective consent procedure with the doctors; i.e., we sought their prospective written consent at the start of the study, but we also asked them to confirm their consent after each recording involving them. Written informed consent was provided by all the doctors, patients, and companions present in the recordings examined in this study.

When recruiting patients, companions, and doctors, we described the study as examining communication between experienced doctors, patients, and patients’ companions (when present). We also told them that the study would concentrate on communication that shows empathy, sensitivity, compassion, and that gives people opportunities to be involved in making plans and decisions about their care. This was because our project had a wider scope, extending beyond the inquiry reported here, and aimed at characterising several domains of communication in palliative and EoL care (other lines of analysis are currently examining empathic communication, decision-making, and pain management). Because we provided a broad description of the topics we were interested in and did not specifically mention communication around EoL, patients, companions and doctors did not have heightened awareness that EoL was a topic that we wanted to explore. In other words, the EoL conversations examined in this report were not triggered by our consent procedure.

We removed all identifying information (person and place names) from the transcripts reproduced in this paper; specifically, in the transcripts we refer to the participants as patients (“Pat”), companions (“Com”), and doctors (“Doc”). We pseudonymised all references to persons and places in the transcripts.

### Patient and carer involvement

We carried out a preliminary exploration of the acceptability of video-recording consultations in the hospice environment; this involved interviewing hospice patients, carers, and members of the care team (report in preparation). Their views fed into the design of our study protocol, specifically on how to make the video-recording process as unobtrusive as possible, and on how to design the recruitment process (e.g. to avoid the risk of coercion and ensure informed consent). Carer representatives were part of the study advisory group and provided feedback on draft versions of the recruitment materials (informed consent form and information sheet) and on the topical foci of our analysis. Patients and carers who took part in the study were offered the opportunity to watch (or listen to) the recording of their consultation and could also request a copy of the study report.

### Analysis

CA is a research approach that examines how participants accomplish social actions, that is, how they do things through talk and visible conduct (e.g. gestures, gaze). [26–29] It involves analysing participants’ actions (e.g. asking questions, making requests, offering assistance), the different ways in which they can implement those actions (e.g. through different types of utterance as well as non-verbal means [33]), and the differential consequences that implementing actions in different ways can have on the unfolding interaction. [34, 35] This approach was especially suited to the present study where we aimed to describe some communication practices that doctors use and their implications for EoL talk.

CA allows examination of the norms that shape social interaction and thereby to understand why people communicate in the ways they do. [28] Relevant to the study reported here is prior CA work demonstrating that people in informal, domestic settings systematically treat some conversational topics as delicate. For instance, people perform special conversational work to enter and to leave talk about personal “troubles”. [36, 37] A study on how people announce another person’s death in informal telephone conversations found that discussion about someone’s death is regularly followed by a shift to more positive commentaries (termed “bright side sequences”). [38] These studies suggest that some topics require special work to be broached and to be terminated. In our study, we also use CA to identify a norm that appears to constrain the organisation of talk about dying, specifically in the form of a *preference* for patients to volunteer EoL considerations rather than doctors to explicitly ask about these. Importantly, in CA “preference” does not refer to a personal or psychological preference for some interactional outcome; rather, it designates norms which people take into account when constructing their actions. [39–41]

Finally, CA relies on proof procedures whereby the analysis of actions and practices is grounded not in researchers’/observers’ post hoc interpretations of what goes on, but instead in people’s local, observable understandings of those actions and practices. [42, 43] This means that claims about what action is being performed are solely based upon tracking (via recordings) how people demonstrably interpret particular practices as implementing precisely that type of action. This analytic proof procedure was particularly apt for examining whether doctors treat parts of patients’ talk as containing cues relevant to EoL matters; it allowed an examination of doctors’ own understandings, displayed in real time, in the course of their interactions with patients. [42]

To analyse doctors’ practices for engaging patients in EoL talk, we employed the following analytic steps, which are conventional in CA. (1) We watched/listened to the recordings alongside verbatim transcripts to identify instances of EoL talk. (2) We isolated doctors’ communication practices that were recurrently followed by movement into EoL talk, and we identified data-internal evidence that the people in the recordings observably treated these practices as contributing to courses of action that promoted EoL talk (e.g. patients’ responses raised EoL matters and, when they did not, doctors further pursued responses that raised EoL). For this study we focused on one particular practice which we term *elaboration solicitation* because it was the most frequently used practice that doctors used to promote EoL talk. Although we acknowledge that doctors used other practices to promote EoL talk, these need separate treatment in future reports. (3) We examined in detail the properties of elaboration solicitations by returning to the recordings, watching them and making detailed transcripts to facilitate examination of aspects of speech delivery (intonation, emphasis, and pace) and temporality (silences, overlaps). [44, 45] We also transcribed visual aspects of communication (e.g. gestures and gaze direction). S2 File describes the transcription conventions we used (these are conventional in CA). (4) We proceeded via detailed description of individual episodes and comparison of multiple episodes in order to identify and describe recurrent patterns and to elucidate their consequences.

MP performed the bulk of the analysis; regular meetings were held with RP to check emerging analyses. Following a consolidated procedure in CA, we held several meetings with VL and experts outside the research team to jointly analyse segments of the recordings which were representative of the communication patterns examined here. This enabled us to check, extend, and refine our emerging analyses.

## Results

For the full study we recorded 43 consultations with consecutive, eligible patients and 5 doctors; for 37 of these, all parties recorded gave written consent for retention and analysis of the recording (33 video, 4 audio). 14/37 consultations contained EoL talk involving 3 doctors, 14 patients and 9 companions in 11 hours of recordings (Table 1).

**Table 1:** Characteristics of the participants.

Patient condition	2 Motor Neurone Disease, 1 Multisystem Atrophy, 1 Heart failure + osteoporosis, 4 cancer, 2 COPD, 1 Gastrointestinal Stromal Tumour, 1 Bronchiectasis + Emphysema + collapsed lung, 1 pulmonary fibrosis, 1 sensorimotor neuropathy
Patient gender	8/14 Male; 6/14 Female
Presence of companions (partner, relative or friend)	7 consultations: 1 companion; 1 consultation: 2 companions; 6 consultations: no companion present
Doctor’s professional role	3 experienced palliative medicine consultants: 2 female, 1 male
Length of the consultations	23–75 minutes (mean 48)
Type of appointment	11 outpatient, 2 day therapy clinic, 1 inpatient
First or follow up appointment	4 first appointments; 10 follow-up appointments

### Practices doctors use to promote EoL talk

The practice most frequently used by doctors to promote EoL talk was to solicit (i.e. promote) elaboration of a part of the patient’s previous talk, or a part of a companion’s talk (N = 48). We term these *elaboration solicitations*. Other practices used by doctors included initial exploratory questions (N = 33; e.g. “Do you feel anxious about things Ian?”, Extract 8, line 1 –all data extracts are provided in S1 File), hints or suggestions of EoL matters (N = 16; e.g. “You’re not stupid. You know what that means, you know, it means that actually people then die”), and proposals to discuss EoL matters (N = 9; e.g. “I think it would be helpful to talk about things and sometimes a bit of rainy day planning isn’t all doom and gloom but it is having your kind of plans in place just in case”).

**Fig 1:**
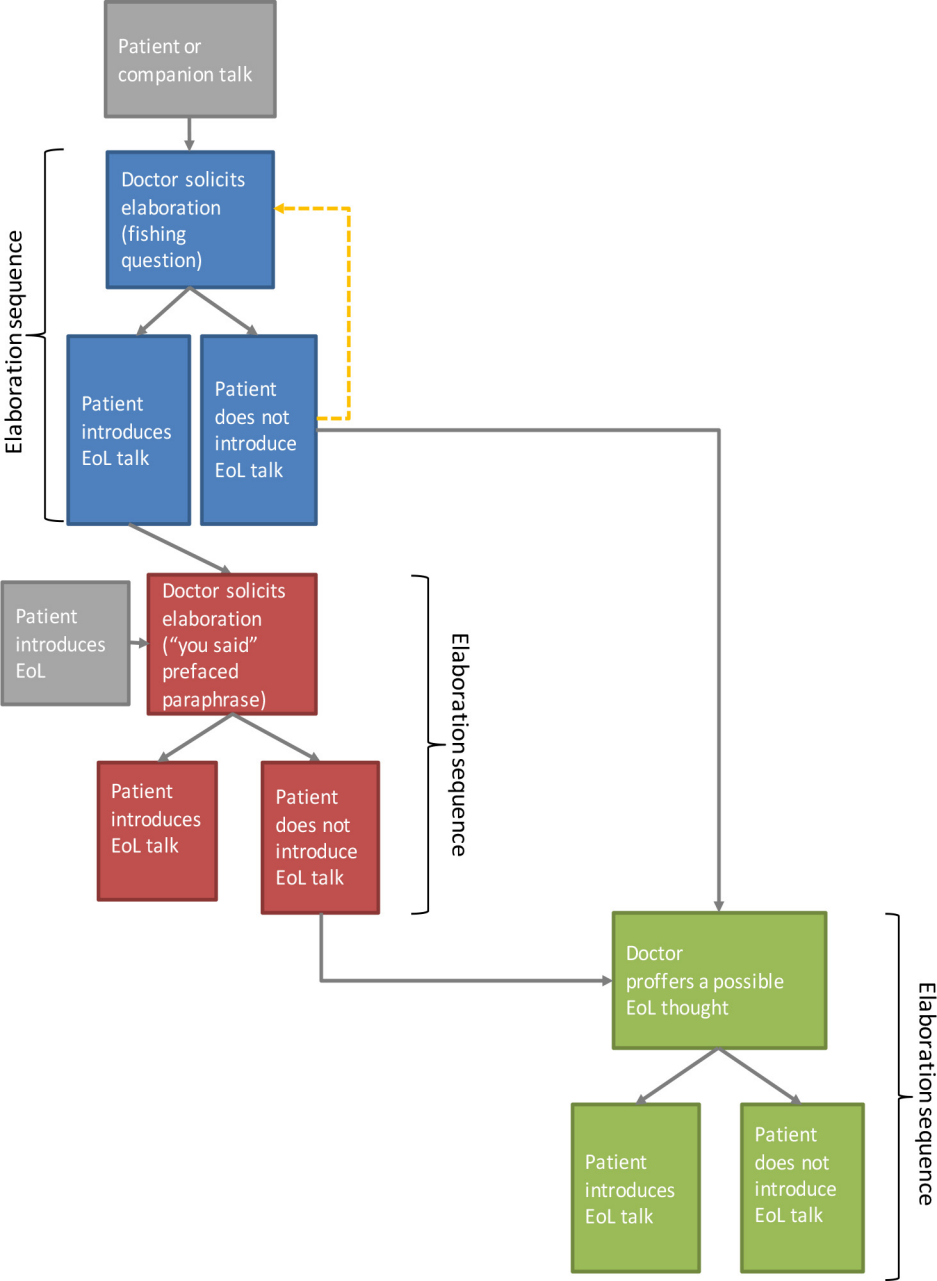
Structure of elaboration sequences.

With elaboration solicitations, doctors launch an *elaboration sequence* (on sequence, see [28]) formed of two actions: (1) the doctor’s elaboration solicitation, and (2) a patient’s (or companion’s) response. We identified 48 elaboration sequences. They occurred both in first visits (N = 4) and follow up visits (N = 10), and their form and functioning did not observably differ across first and follow up visits. The number of times that the doctors used elaboration solicitations within a single consultation varied from 1 to 6 (M = 3.4; SD = 1.8). The following sections analyse the three types of elaboration solicitation and how they operate to promote EoL talk. The three types are: (1) *Fishing questions*, e.g. “And when the pain’s bad and you start to feel a bit panicky, can you remember what’s going through your mind at that time?” (Extract 1, lines 33–36); (2) *“You said”-prefaced paraphrases*, e.g. “so coming back to what you were saying before for a second Lynn, part of it is the fear of what might happen?”, (Extract 6, lines 12–14); and (3) *Proffering possible EoL thoughts*, e.g. “do you worry about what’s coming?”, (Extract 9, line 21). Fig 1 illustrates the structure of elaboration sequences. The analyses that follow refer to data extracts provided in S1 File. For illustrative purposes and for space considerations, in this paper we present examples where doctors direct their elaboration solicitations to patients, however our data also includes cases where they direct them to patients’ companions.

### Fishing questions: Providing opportunities to introduce possible EoL considerations

One practice doctors used to seek the elaboration of something said by the patient (or occasionally a companion) was a *fishing question* (Fig 1, blue section). [46–48] In fishing questions, the doctor follows up on a patient’s description of a problem or difficulty (e.g. pain, sleeplessness, mobility issues) by asking about the patient’s associated thoughts, views and concerns (e.g. “Do you think that’s all around your breathing getting worse or something else?”, Extract 2), or about thoughts the patient has when experiencing symptoms (e.g. “When the pain’s bad … can you remember what’s going through your mind a that time?”, Extract 1). So, fishing questions are selective in that they do not just ask ‘tell me more?’ but instead ask the patient to elaborate in terms of their thoughts, views, and concerns associated with specific matters; and these matters are such that further talk on them *could* lead towards a subsequent focus on EoL considerations–i.e. reports of thoughts, concerns, and views about the prospect of the patient’s deterioration and death. Whilst selective in this particular way, fishing questions are also *open* in that they ask what patients’ thoughts, views, or concerns are rather than proposing particular thoughts, views, or concerns (a much more selective type of question, e.g. “Do you worry that you’re going to die when that happens?”, is examined in the section “Proffering a possible EoL thought”, below). Fishing questions thus provide *opportunities* for patients to introduce EoL considerations, and therefore to engage in EoL talk without it having been overtly solicited–hence the term “fishing”. At the same time, they make it possible for the patient not to engage in EoL talk by responding instead with other, non-EoL related considerations, and to do so without having to actually reject a doctor’s invitation to discuss EoL matters–precisely because the doctor has not produced such an explicit invitation.

Extract 1 is a transcribed episode from an outpatient consultation involving a patient with cancer and his brother (all extracts are in S1 File). The brother reports that, when the patient experiences pain, his breathing gets worse, he panics, and he calms down by using an inhaler (lines 1–16). The patient downplays the seriousness of the problem and confirms that the inhaler calms him (lines 21–31). The doctor asks the patient the *fishing question* “what’s going through your mind at that time?” (lines 33–36), which seeks his elaboration on his brother’s description of pain and panic. This is an open question about the patient’s thoughts, which does not suggest what those thoughts might be; it leaves it open to the patient either to report thoughts about the prospect of dying or other kinds of thoughts–either would be a relevant and appropriate response.

Another example of a fishing question can be seen in Extract 2, a transcribed segment of a day therapy consultation with a patient with severe heart failure and osteoporosis. The doctor’s fishing question “Do you think […] that’s all around your breathing getting worse or something else?” (lines 9–10) asks the patient to elaborate on the mood problem she has mentioned. Although the doctor uses what is grammatically a yes/no question, the action this implements is actually an open question about what might underlie the patient’s mood change, aside from her current main problem (shortness of breath). The fishing question does not however suggest what that might be. Thus, it is open to the patient to either introduce EoL or non-EoL concerns–both are relevant, legitimate possible responses to the question.

A further example of a fishing question can be seen in Extract 3, which is a transcribed episode from an inpatient consultation with a patient who has a gastrointestinal stromal tumour. The fishing question “Have you had any thoughts about it?” (line 20) asks the patient to elaborate on a back pain complaint. Once again, although grammatically the doctor’s question has a yes/no format, it actually implements the action of asking an open question about “why it’s come on” (a concern that the patient himself raised at line 12). The patient can relevantly respond to the fishing question by linking the pain problem to his terminal diagnosis or by reporting other types of thought (and indeed he does at lines 22–25 by introducing a complaint about his GP).

Patients sometimes introduce EoL considerations in response to doctors’ fishing questions; this provides evidence that patients do, at least sometimes, treat these questions as creating opportunities for EoL talk. Furthermore, when patients’ responses to fishing questions do not introduce EoL considerations, doctors recurrently pursue further responses (Fig 1, orange dotted arrow) until patients introduce EoL considerations or actually voice unwillingness to do so; this provides some evidence (albeit post-hoc) that doctors may have produced the initial fishing question to give patients a first opportunity to introduce EoL considerations. In Extract 1, when the doctor poses a fishing question about what is going through the patient’s mind during coughing bouts, the patient’s response focuses on symptoms (“it’s just cough”, line 38), and therefore does not introduce EoL considerations. The doctor proceeds to issue two new versions of the fishing question: “What do you think it’s gonna happen?” and “What are you thinking?” (lines 42, 45 and 47). Following a pattern that recurs in the consultations we recorded, the doctor modifies his original fishing question and narrows down its focus, possibly to cue the patient to EoL considerations in a subtle way. In this particular case, he narrows down the focus of the question to thoughts/concerns beyond the patient’s immediate experience of pain and panic (“What do you think *it’s gonna happen*”, line 45), but, again, without explicitly suggesting death as a possible concern. This gives the patient the opportunity to be the first to bring an EoL consideration into the consultation, and indeed he does so by reporting that he has thoughts about dying when he gets pain: “Oh (an’) this is it now” (lines 51–53).

In another example, Extract 4, from later in the same consultation as Extract 3 (where the patient with the gastrointestinal stromal tumour did not introduce EoL considerations in response to the doctor’s “Have you had any thoughts about it?” fishing question), the doctor pursues a further response to her earlier fishing question about the patient’s pain problem. She narrows down the focus of the question to the patient’s biggest concern, thus possibly cueing the patient to EoL considerations in a subtle way: “So just going back to you worrying about your back pain […] are you able to share what’s worrying you *most* at the moment?” (lines 1–7). Despite the doctor’s repeated attempts at eliciting a different response (lines 10 and 22–23) the patient only reiterates his present concern about back pain (lines 12 and 16) without introducing EoL considerations (lines 24 and 26). Later in the same consultation (Extract 5), the doctor further pursues the patient’s thoughts; following the pattern observed throughout the consultations, she re-specifies and narrows down the focus of her questions to increasingly cue the patient to EoL considerations. In this particular case, she adjusts her question to elicit thoughts beyond the patient’s immediate concerns: “Do you have any sense in your mind what’s happening with your […] with your disease, with your tumour, […] what do you think?” (lines 1–4, 16). The patient’s responses clearly convey his unwillingness to think about the disease: “I try and shut my mind off it. […] I don’t want to know about it.”, (lines 18 and 28). These responses also display his understanding of the doctor’s questions as pursuing EoL talk (“Don’t want to talk about it.”, line 38) and convey that engaging in EoL talk is definitely not on his agenda (as he notes, “I’m a stubborn person”, line 46). The doctor accepts this (line 53) and subsequently starts to bring the consultation to a close (data not shown).

To summarise, in some cases where patients do not introduce EoL considerations in response to doctors’ fishing questions, doctors produce additional fishing questions which add some restrictions in terms of what would count as a relevant response, until patients either introduce EoL considerations or display unwillingness to do so. This provides some evidence that doctors use fishing questions early, and strategically, in an attempt to engage patients in EoL talk. We turn next to consider the relationship between fishing questions and “patients’ cues”.

So far, we have shown evidence suggesting that doctors use fishing questions strategically to give patients opportunities to introduce EoL considerations. [49] We further observe that patients’ responses often convey *possible* rather than definite EoL considerations; [50] for instance, patients use allusive or euphemistic expressions (“Oh this is it now”, Extract 1) and descriptions of emotional states (“Fear”, Extract 2), which *only possibly* refer to their future deterioration and death. Staying with the latter example, the patient describes a state of “fear”, and later associates it with “the reality of knowing what’s happening” (lines 18–19); this might refer either to her experience of breathlessness or to the prospect of her further deterioration and death. Doctors recurrently solicit patients’ further elaboration of those expressions (this is examined in the next section), and this strongly suggests that doctors may be treating those expressions, and the topics that they introduce, as opportunities to broach EoL talk. Does this also mean that doctors are treating those expressions as “cues” relevant to EoL concerns? [51] One difficulty associated with using the term “cue” is that it can be understood as assuming that the patient is, on purpose, intentionally, hinting at EoL concerns they have; and that the doctor is assuming, in the course of the consultation, that those parts of the patient’s talk do indeed suggest an intention to discuss EoL. Our analyses do not support these conclusions. For instance, although the subsequent elaboration of the patient’s expressions in Extract 2 (“fear” and “the reality of knowing what’s happening”) leads to more explicit EoL talk later in the consultation (Extract 6, examined in the next section), this does not demonstrate that the patient meant those expressions to convey possible EoL considerations in the first place. Also, the fact that the doctor later solicits further elaboration of that expression (“so coming back to what you were saying before […] part of it is the fear of what might happen?”, Extract 6, lines 12–14) does not demonstrate that he interpreted that expression as indicating a patient’s need or desire to discuss EoL. The doctor may have simply seen in the patient’s expression of fear an opportunity to engage her in EoL talk, irrespective of the patient’s underlying intentions.

We propose that it is not necessary to speculate about patients’ and doctors’ motivations in order to understand the process of how EoL talk is initiated. Our findings suggest that EoL talk is interactionally generated by inviting patients’ elaboration of parts of their previous talk–regardless of patients’ intentions underlying the production of those expressions. When patients respond with possible EoL considerations, doctors subsequently invite them to further elaborate on those expressions. This is examined in the next section.

### “You said”-prefaced paraphrases: Promoting further elaboration of a possible EoL consideration

When patients mention possible EoL considerations, at some time later in the consultation, doctors recurrently invite patients to expand on these. This is a further step in promoting a progression from possible EoL considerations to actual EoL talk (Fig 1, red section). Doctors do this through a second form of elaboration solicitation, which we term a *“you said”-prefaced paraphrase*; this is an utterance that proposes a version of something that the patient has said in a previous part of the consultation. The utterance contains a component that refers back to what the patient has said earlier, such as “you said”, “you mentioned”, “you were saying”, and the like. For convenience, we amalgamate these forms into “you said”. [52] The “you said” component is followed by a paraphrase, i.e. a version that offers the doctor’s understanding of that earlier talk. This practice has similar properties to what previous studies have termed a “formulation”, i.e. utterances that show understanding of something the patient has said by proposing a version of it. [53–55] Like formulations, “you said”-prefaced paraphrases encourage the patient to confirm (or disconfirm) the doctor’s conveyed understanding of the patient’s earlier talk, and they promote an elaboration of that talk. Unlike formulations, “you said”-prefaced paraphrases are not used immediately after the patient’s target talk, but refer back to it at some time later in the conversation. Also, “you said”-prefaced paraphrases may be a way of doing the action of “summarising” as described in some guides to communication skills training–although research should be conducted to support this speculation. [56]

Unlike fishing questions, which cast a wide net in terms of the responses the patient could give, “you said”-prefaced paraphrases do not provide opportunities for introducing *new* EoL considerations, but instead focus in upon one specific, and already expressed, possible EoL consideration and encourage its further elaboration. In Extract 2 the patient produced a possible EoL consideration: “the reality of knowing what’s happening” (lines 18–19); then the talk moved to other matters. Later in the same consultation (Extract 6) the doctor uses the “you said”-prefaced paraphrase “so coming back to what you were saying before […] part of it is the fear of (0.3) what might happen?” (lines 12–14). This utterance refers back to what the patient said earlier in Extract 2 and proposes a version of it. In what he says here, the doctor does not overtly ask the patient to say more and he does not suggest what the nature of the fear might be. At the same time, the doctor narrows down the focus to concerns beyond the experience of breathlessness (“what *might happen*”), hence possibly cueing the patient to EoL considerations in a subtle way. This provides the patient with an opportunity to elaborate on her earlier reference to fear in EoL terms, and she does indeed opt to do so: “I’ve never been frightened of dying […] until just lately” (lines 15–19). The outcome is to bring EoL clearly into focus without the doctor overtly having asked the patient about it.

The example in Extract 7 is different in that it shows a patient opting not to respond in terms of EoL concerns. Earlier in the consultation (in data not shown), in the course of complaining about the ineffectiveness of some pain medication, the patient has said that sometimes he feels like “throwing the towel”, possibly alluding to EoL thoughts; the talk then shifted to other matters. As Extract 7 begins, the patient has returned to complaints about his medication (lines 1–2). The doctor uses a “you said”-prefaced paraphrase that retrieves the patient’s earlier possible allusion to EoL thoughts (lines 8–9). Whilst she produces a version of that expression (“You mentioned about a feeling of throwing in the towel”) she does not make any suggestion about what its meaning might be. Following this the patient clarifies the expression in non-EoL terms (“stop taking the pills”, lines 11–12); the doctor gives the patient an opportunity to revise (“Stop taking the pills. That’s what you mean by that?”, lines 13–14), but he confirms the non-EoL meaning of that earlier expression (“Yeah”), which the doctor then accepts (“Okay”). This shows that “you said”-prefaced paraphrases share with fishing questions the property of leaving it to patients to either engage in EoL talk or not; introducing non-EoL considerations allows patients not to engage in EoL talk, and to do it without having to explicitly decline or resist an explicit invitation to do so–precisely because the doctor has not produced such an invitation.

In sum, “you said”-prefaced paraphrases retrieve a possible EoL consideration already mentioned by the patient and encourage its further elaboration by the patient. Doing so can enable the pursuit, the bringing back into focus, and the consolidation of EoL considerations as a topic for the conversation. At the same time, it is open to patients to respond to this kind of utterance in non-EoL terms.

### Proffering a possible EoL thought: Pursuing EoL talk more overtly

Fishing questions and “you said”-prefaced paraphrases are open elaboration solicitations in that they promote patients’ elaboration of parts of their previous talk without suggesting EoL as a direction for that elaboration. This means that it is left to patients to either introduce EoL considerations or not; it also means that patients can avoid engaging in EoL talk by volunteering non-EoL considerations, which is a perfectly relevant response to fishing questions and “you said”-prefaced paraphrases. However, there are circumstances where doctors do suggest EoL as a direction for the elaboration. Rather than asking about patients’ thoughts in general, doctors *proffer a possible EoL thought*.

In Extract 8 (from the same consultation as Extract 7), in response to an initial question about anxiety (line 1), the patient, who has motor neurone disease, talks about the death of another hospice patient and how this “brought everything back home”, a phrase hearable as euphemistically referring to his thoughts/concerns about his own death (lines 11–12). With her “mm” continuer (line 14), the doctor gives the patient an opportunity to say more [57], and, after a substantial silence, the patient comments on the circumstances of the other patient’s death without further elaborating on the impact that this event had on himself (“As it seems so unexpected as well”, line 18). After the patient’s displayed difficulty in forwarding the topic (“.ksHHH (0.3) tklk and that (1.6) and = hh (2.2) hhhh (0.3) don’t know.”, lines 20–21) the doctor launches an elaboration sequence by proffering a possible EoL thought: she asks whether the other patient’s death made the patient think about his own death (line 22). Whilst the doctor uses a more overt approach to raising EoL, she mitigates it by avoiding explicit mention of the patient’s death: “Did it make you think *about you*”. So, even though the doctor narrows down the terms in which the patient could relevantly answer the question, she nevertheless leaves it open to the patient to be the first to mention “dying” within the consultation (and he indeed does so: “I thought well you’re going to die one day”, lines 26–27).

We recurrently found that, when doctors proffer possible EoL thoughts, this happens after they have already created multiple opportunities where patients could volunteer EoL considerations (via fishing questions and “you said”-prefaced paraphrases) but where patients have not taken up those opportunities to do so (Fig 1, green section). By switching to this more presumptive type of elaboration solicitation, doctors pursue EoL talk more overtly. This is illustrated in Extract 9, which comes from an outpatient consultation involving a patient with cancer and his wife. Earlier (in data not shown) the doctor used several fishing questions, asking the patient to share his thoughts about different aspects of his medical condition, to which the patient did not respond with any EoL considerations. At lines 12–19, the doctor summarises the problems reported by the patient which, taken together, strongly suggest that he is on an EoL trajectory, and she uses them as a basis for now asking whether the patient worries about “what’s coming”, which euphemizes dying. This launches an elaboration sequence where, unlike the cases in the previous sections, the doctor lays out EoL as a direction for the elaboration. At the same time, she cites as grounds for asking the question several aspects of the patient’s condition, which he reported during the consultation: cyanosis, a terminal diagnosis given by a respiratory consultant, and receiving an experimental treatment that did not work (lines 12–15). [58] Furthermore, her listing of these matters works to reduce the patient’s options for referring to these when he answers her question. Despite an initial negative answer (line 23), the patient introduces, albeit in vague terms, an EoL matter, i.e. his prognosis (lines 25–32).

One key difference between the type of elaboration solicitation examined here and the ones analysed in the previous sections (fishing questions and “you said”-prefaced paraphrases) is that they place more constraints on what counts as a relevant response. When doctors use open elaboration solicitations (fishing questions and “you said”-prefaced paraphrases), patients can avoid engaging in EoL talk by reporting non-EoL concerns, that is, through *affirmative* responses. By contrast, if patients select not to engage in EoL talk, they have to do it through a negative response (e.g., hypothetically, “No” in response to “Do you worry about what’s coming?” in Extract 9). This is arguably more difficult to do because it entails avoiding aligning with the project embodied in the doctor’s question; [28] also, it entails disconfirming having EoL concerns and patients might be reluctant to do so if this does not hold true. Extract 10 is a final example of proffering an EoL thought. This is from a consultation where, despite having disclosed that sometimes he has EoL thoughts (Extract 1, lines 51–63), the patient’s talk later focused on his eagerness to receive treatment by enrolling in a clinical trial (referred to in Extract 10 at lines 1–2). This optimistic orientation created a context in which it was difficult for the doctor to promote EoL talk as an elaboration of the patient’s own talk using fishing questions or “you said”-prefaced paraphrases. Instead, after acknowledging the patient’s hope for treatment (“So that’s one thing is hoping that the Royal will have treatment that’ll work”, lines 6–8), the doctor proffers a possible EoL thought. In this case, he does so by raising a contrastive hypothetical scenario where the patient cannot get treatment, and by asking for the patient’s thoughts about that scenario: “do you ever wonder what will happen if they don’t have treatment that works?” (lines 11 and 13). [48] After an initial negative answer (line 14), the patient elaborates on his expectation of receiving treatment (23–39), therefore maintaining an optimistic orientation. However, he then introduces his deteriorating condition and his thoughts about the possibility of dying (lines 42–52). The doctor uses this as an opportunity to pursue more EoL talk by referring back to the patient’s earlier mention of EoL thoughts (lines 54–55). As in the previous examples, at lines 11 and 13 the doctor pursues EoL talk more overtly (by explicitly asking whether the patient has thoughts about the possibility of not recovering), however he does not explicitly mention “dying”. Additionally, he mitigates his more presumptive elaboration solicitation by referring back to–and therefore by grounding his questions on–things that the patient himself has said earlier in the consultation (“Cause you said when the coughing was bad, sometimes you wonder if it might be then?” lines 54–55).

In sum, by proffering possible EoL thoughts doctors take a less participatory and more presumptive approach to promoting EoL talk and do so in a way that ask patients whether or not they have EoL thoughts/concerns. However, doctors also mitigate it in two ways: they do not overtly mention “dying”/“death” (Extracts 8–10), and they draw on things that patients have themselves said in the course of the consultation in order to suggest–albeit indirectly–the patients may already have EoL thoughts in mind (Extracts 9 and 10). We suggest that doctors’ actions are shaped by an overall preference for patients to introduce EoL talk as opposed to introducing it on their behalf. As noted above (in the Analysis section), in CA preference does not refer to a personal or psychological preference for some interactional outcome; rather, it designates norms that shape how people communicate with one another. Along these lines, our findings strongly suggest that experienced doctors withhold introducing the topic of EoL in places where they could do so, and instead, they use forms of talk that leave it to the patients to take the initiative to introduce EoL considerations; only after patients have repeatedly forgone volunteering EoL considerations do doctors use questions that suggest the presence of EoL concerns.

## Discussion

We examined how experienced doctors engage terminally ill patients in EoL talk in palliative care consultations. By using open elaboration solicitations (fishing questions and “you said”-prefaced paraphrases), doctors provide opportunities for patients to introduce possible EoL considerations and, subsequently, to further elaborate on them. Some consequences are, firstly, that EoL talk emerges as a continuation of patients’ previous talk rather than as a new or disjunctive topic; secondly, that EoL talk is produced in a participatory way as the result of soliciting an elaboration of patients’ talk in open terms, for example by inquiring about what the patient’s thoughts/concerns are regarding their condition, rather than suggesting what those thoughts/concerns may be; and, thirdly, that patients can avoid engaging in EoL talk by relevantly reporting a non-EoL consideration. By leaving it to patients to report EoL or non-EoL considerations, doctors can avoid being seen as pushing patients to engage in (unwanted) EoL talk, and patients are not put in the position of having to explicitly refuse to do so. Furthermore, by not explicitly asking whether patients have EoL thoughts/concerns, doctors avoid the risk of receiving a negative answer at the start, and this leaves the door open for them to pursue EoL talk in later phases of the consultation. At the same time, doctors balance this participatory approach with several practices that enable them to pursue EoL considerations in circumstances where patients do not immediately volunteer them, as follows. First, they create repeated opportunities for patients to introduce EoL considerations by using multiple fishing questions in the course of a single consultation (e.g. as seen in Extracts 3 and 4). Second, doctors progressively adjust the design of their elaboration solicitations to ask patients about their most serious concern (e.g. “What’s worrying you *most*”, Extract 4) or about concerns beyond their main, current problem (e.g. “what do you think [is] *gonna happen*”, Extract 1), hence possibly cueing patients to EoL in a subtle way. Third, in some consultations, when patients do not volunteer EoL considerations in response to these participatory practices, doctors switch to a more presumptive form of elaboration solicitation, which suggests EoL as a direction for the elaboration, and thus more forcefully pursues EoL talk. The fact that doctors more overtly pursue EoL talk only later in the consultations suggests that their practices are shaped by a preference for EoL considerations to be introduced by patients rather than doctors.

Our study has both strengths and limitations. A strength is that we examined in fine grained detail recorded real-life interactions between experienced doctors and terminally ill patients. Using CA allowed us to examine how doctors and patients display understandings of each other’s actions in real time, and how their actions are constrained and enabled by norms that underpin their communication. This approach was especially suited for a study of communication in palliative and EoL care. Previous research in this area has relied on retrospective descriptions of HCP-patient interaction (such as through interviews). Although that research has delivered important insights on what counts as good communication for HCPs, patients, and their companions, it has not been able to analyse how this good communication is actually implemented. Our study fills this gap and contributes to an emerging strand of research on the organisation of EoL talk across different healthcare settings. [14, 48, 59–61] One limitation is that we recorded at a single English hospice, and our findings are based on the practices of three consultants. This necessitates caution in generalizing to other settings and indicates that further research is required to establish whether the practices observed here are employed elsewhere. Some other limitations to the transferability of our findings are that the consultants in this study had years of training and experience in engaging patients in EoL talk, and that the consultations analysed in this study lasted 48 minutes on average. The presence of less trained clinicians and constraints on the duration of consultations may influence how these conversations unfold in, for instance, hospitals or GP settings. Also, the patients in this study may have somehow been primed to discuss dying because the conversations happened in a hospice, whereas hospital, GP surgery or home may present different conditions. The study only recruited patients who had mental capacity and who spoke and understood English; this does not cover the full spectrum of terminally ill patients who receive EoL care. Despite these limitations we argue that the communication practices examined in this study are available for anyone to use and are therefore potentially transferable to other settings.

Future research should address how clinicians engage patients in EoL talk in different settings; other conversational practices that doctors use to promote EoL talk; whether some practices for engaging patients/companions in EoL talk are more effective than others in terms of actually resulting in EoL talk; how patients and doctors transition to planning for future care once the topic of dying has been raised; and how patients and their companions perceive EoL conversations and what they understand to have been communicated.

Our study has considered the notion of patients’ cues in a radically different way. It has been recommended that doctors promote EoL talk by responding to patients’ cues relevant to EoL concerns. [8, 17–21] However, previous studies only examined patients’ cues using a post-hoc and external analytic approach. [24, 25] In contrast, by using CA, we asked whether doctors themselves observably treat parts of the patients’ in-consultation talk as containing cues relevant to EoL concerns. We found evidence to support that doctors treat parts of patients’ talk, and the topics that they introduce, as opportunities to broach EoL talk. However, we did not find evidence that they treat that talk as alluding to a patient’s intention to express EoL concerns–as the term “cue” suggests. We propose that it is not necessary to speculate about patients’ motivations to understand the process of EoL talk initiation. [62, 63] Observably, doctors’ elaboration solicitations prompt patient responses that introduce possible EoL considerations; further elaboration of these recurrently leads to engaging in EoL talk. We therefore suggest the term “possible EoL consideration” should be used as a descriptively more accurate substitute for the term “cue relevant to EoL concerns”. Additionally, the notion of “responding to cues” is too narrow because it can suggest that doctors immediately respond to patients’ possible EoL considerations. We demonstrated that doctors may retrieve and solicit the elaboration of possible EoL considerations in later parts of the consultation.

We argue that the practices examined in this paper can enable HCPs to navigate the dilemma of EoL talk initiation. Previous research suggests that HCPs can be reluctant to raise the subject of dying with patients for fear of causing them harm. [64] This can lead HCPs to wait for patients to raise the topic first, sometimes resulting in EoL not being discussed at all. [65] Thus, some studies and guidelines recommend that HCPs explicitly ask patients whether they wish to discuss dying. [15, 66] However, there is also concern that doing so through the use of standardised tools and protocols can encourage a scripted, routinized approach to EoL conversations that supplants more person-centred and relational care. [5, 67] In this debate, HCPs can be caught in a tension between the expectation that EoL talk is actively promoted, and the expectation that this is done in a way that is sensitive to patients’ communication needs. [5, 8, 68–71] The communication practices we describe here can enable HCPs to navigate this dilemma. By providing multiple opportunities to volunteer EoL considerations, HCPs can actively enable patients to bring up the topic of dying, rather than passively waiting for them to do so and without, at the same time, overtly inviting EoL talk.

## Supporting Information

S1 File Data extracts. (PDF)

S2 File Transcription conventions. (PDF)
