# Supplementary material for: Engaging Terminally Ill Patients in End of Life Talk: How Experienced Palliative Medicine Doctors Navigate the Dilemma of Promoting Discussions about Dying
Source: PLoS One. 2016 May 31;11(5):e0156174. doi: 10.1371/journal.pone.0156174 (PMC4887020; doi:10.1371/journal.pone.0156174)
Supplement: S1 File — (PDF) [file pone.0156174.s001.pdf]

Supporting information to Pino M, Parry R, Land V, Faull C, Feathers L, and Seymour J (2016). Engaging terminally ill patients in end of life talk: How experienced palliative medicine doctors navigate the dilemma of promoting discussions about dying. *PLOS ONE*.

S1 File. Data Extracts

Extract 1. Hospice outpatient consultation with a male patient – diagnosed with neck tumour and lung metastases – and his brother companion.

```
VERDIS31 15.13 VT310 EL31.2 MP line 15
Dr D
Time into the consultation: 14 min

01 Com:      You know when he (0.2) he'[s bad you know the pain
02 Doc:                                 [Mm.
03           (0.4)
04 Doc:      Y[eah.
05 Com:       [(A:n::')/(The:n::) (1.2) then his breathing
06           and everything goes.
07 Doc:      It's bad [altogether.
08 Com:               [And then he panics.
09 Doc:      Yeah.
10           (0.2)
11 Com:      Then he he has to have a (0.4) you know (i- uh the) (0.3)
12 Pat:      Inhale[r I have ↑it
13 Com:            [just-
14 Com:      just to
15 Doc:      Ye[ah.
16 Com:        [calm him [down.
17 Pat:                  [Mm.
18 Com:      A[nd
19 Doc:       [Okay.
20 Com:      'n the[n
21 Pat:            [It's just (the) ↑cough it's just I can't
22           ↑cough (tch)
23 Doc:      ↑Sure
24           (0.6)
25 Pat:      So that calms me down. Then I'm okay I-
26           and then I have [medicine so .hhh[h
27 Doc:                      [Mm.             [Yeah.
28 Pat:      that will (        in) within half an hour,
29           so then I'll calm down.
30           (.)
31 Pat:      (Then) it's fine.
32           (0.3)
33 Doc: ->   And when the pain's ba::d and you f-
34      ->   start to feel a bit panicky,
35      ->   .hhh can you- (0.2) can you remember
36      ->   what's going through your mind [at that time?
             {Doc: circular hand gesture at the side of his own head}
37 Pat:                                     [fhhhhh ((= sigh))
                                             {Pat: lateral head shake}
38 Pat:      .hh It's just cough, (one (h)a) .hhh it's terrible.
39 Doc:      So cough cough cou[gh,
40 Pat:                        [Cough cough cough.
41           .hhh And (0.2) .h
42 Doc: ->   And what [you- what what you-]
43 Pat:               [(oh    and)   my  e]yes goes
44           funny and .hhh I just (0.3)
45 Doc: ->   What do [you think it's gonna happen?
46 Pat:              [I wa-
47 Doc: ->   What's- [what are you thinking.
48 Pat:              [hhhh
```

```
49 Pat:     ( )hhhh ((= sigh))
50 Doc:     It's [difficult to (answer) (    [ )
51 Pat:          [( )hh                      [A very
52          rough tough time? Sometimes I feel like, (.)
53          .hhh "Oh (an') this is it now".
54 Doc:     Awright.
55 Pat:     .hh [('n I)
56 Doc:        [Okay.
57 Pat:     I mean I shouldn't b(h)e s(h)aying,
58          .hhh but sometime I do feel a:: (0.2)
59          .h[h
60 Doc:       [Sometimes you won[der if this is it?]
61 Pat:                         [Yeah    I'm   just] wondering (a)
62 Doc:     [Right?
63 Pat:     [I feel like it's coming now it's coming.
```

Extract 2. Hospice day therapy consultation with a female patent diagnosed with severe heart failure and osteoporosis, and a past history of lymphoma. She is very short of breath.

```
VERDIS16 00,01 VT1 EL16.1 MP line 186
Doctor D
Time into the consultation: 3.47 minutes

01 Pat:      It's not (0.5) a (0.9) a desperate
02           problem or anything like tha:[t.
03 Doc:                                   [No:
04 Pat:      But we don't want it to get that way do  [we   so]: (0.8)
05 Doc:                                               [Okay.  ]
06 Pat:      I'm just telling you tha:: (1.4) uh my mood
07           has dripped- (0.4) dropped a bit.
08           (0.8)
09 Doc: ->   Do you think that's re- (.) that's (.) all around your
10      ->   breathing getting wo:rse or (.) something else?
11 Pat:      tk If you wan'- (that) was fear. I think it's
12           just fear.             {Doc moves head towards the patient}
13           (.)
14 Doc:      Fear.                  {Doc nods}
15           (0.2)
16 Pat:      Just fear.
17           (0.7)
18 Pat:      Just (0.2) the reality of knowing
19           what's happening a:n:' (0.2)
20 Doc:      °Right.°
21 Pat:      not being able to (0.7) ↑do anything about it.
```

Extract 3. Hospice inpatient consultation with a male patient diagnosed with gastrointestinal stromal tumour.

```
VERDIS42 10,37 VT164 EL42.1 MP
Dr F
Time into the consultation: 6 minutes

01 Doc:     Anything e:lse that (0.7) is kind of on your list
02          of (0.6) worries at the moment?
03          (0.6)
04 Pat:     At the mome:nt?
05          (0.2)
06 Doc:     Yeah.
07          (0.9)
08 Pat:     U:::m: (1.7) my back pain.
09          (0.2)
10 Doc:     Okay.
11          (0.7)
12 Pat:     I don't know why it's come o:n.
13          (0.5)
14 Doc:     Right.
15          (0.7)
16 Pat:     It's a:ll down my spi:ne.
17          (0.2)
18 Doc:     Yeah.
19          (1.4)
20 Doc: ->  Have you had any thoughts about it?
21          (0.7)
22 Pat:     Mainly (1.6) my gee pee, ((GP)) (1.3)
23          we:ll (0.3) I don't know,
24          (1.3)
25 Pat:     what he's been doing.
26          (0.8)
27 Doc:     Right.
```

Extract 4. From the same consultation as Extract 3 – male patient diagnosed with gastrointestinal stromal tumour.

```
VERDIS42 12,31 VT214 EL42.2 MP
Dr F
Time into the consultation: 7.53

01 Doc:      So just (.) going back to you worrying about
02           your (0.3) back pain, (0.2)
03 Pat:      Mm mm.
04           (0.6)
05 Doc: ->   Are you able to share what's worrying you mo:st
06           (0.2)
07 Doc:      at the mo[ment?
08 Pat:               [Back pain.
09           (.)
10 Doc: ->   Mm:. But what about it.
11           (0.6)
12 Pat:      It's the absolute mu:rder.
13           (1.4)
14 Doc:      Okay.
15           (.)
16 Pat:      I wish it could go away.
17           (0.7)
18 Doc:      Okay.
19           (1.2)
20 Doc:      You said you're wondering <why> i:t's so ba:d,
21 Pat:      Yeah.
22 Doc: ->   Mm: (.) and have you had any thoughts
23      ->   as to why (0.9) it's so ba:d?
24 Pat:      No:
25           (0.6)
26 Pat:      Just came o:n.
27           (0.4)
28 Doc:      Okay.
```

Extract 5. From the same consultation as extracts 3 and 4 – male patient diagnosed with gastrointestinal stromal tumour.

```
VERDIS42 18,05 VT351 EL42.3 MP
Dr F
Time into the consultation: 13.28

01 Doc:     Do you have any sense in your mind (0.4)
02          what's happening with your (0.9)
03 Pat:     No:
04 Doc:     With your disease, with your tumour,
05          (0.4)
06 Pat:     No:.
07          (0.3)
08 Doc:     °Okay.°
09          (0.3)
10 Doc:     Is it something you think about?
11          (1.0)
12 Pat:     M::::: a lot of the time, (2.6)
13          a lot of the time,
14 Doc:     °Okay?°
15          (1.0)
16 Doc:     And when you think about it, what do you think?
17          (1.3)
18 Pat:     I try and shut my mind off it.
19          (1.4)
20 Doc:     °°Okay? (Right.)°°
21          (1.1)
22 Pat:     I do:.
23          (0.7)
24 Doc:     °°Okay?°°
25          (1.7)
26 Doc:     And why do you think you do that?
27          (1.0)
28 Pat:     (Because I don't wanna) know about it.
29 Doc:     You don't want °(to know).°
30          (0.5)
31 Doc:     Okay.
32          (1.4)
33 Pat:     That's why:.
34          (0.4)
35 Doc:     O:kay.
36          (2.7)
37 Doc:     Okay.
38 Pat:     Don't wanna talk about it.
39          (.)
40 Doc:     °N:o.°
41          (1.0)
42 Doc:     Okay.
43          (1.4)
44 Doc:     HHHHH ((laugh))
45          (0.5)
46 Pat:     I'm a stubbo:rn (0.2) person?
47          (1.8)
48 Doc:     I think we a:ll (0.4) we all cope with our lot
49          in different ways don't we?=And
50          (0.3)
51 Pat:     hhh
52          (1.9)
```

```
53 Doc:     Okay.
```

Extract 6. From the same consultation as Extract 2 – female patent diagnosed with severe heart failure and osteoporosis, and a past history of lymphoma. She is very short of breath.

```
VERDIS16 00,01 VT1 EL16.1 MP line 359
Doctor D
Time into the consultation: 8.30 minutes

01 Pat:      You know and I'm thinking and I'm saying to Michael, (.)
02           I said to Michael “(Oh) please (.) do- don't ge:t upset
03           I'm just telling you this is how it ↑i:s”
04           (0.3)
05 Doc:      Ye:s.
06           (0.6)
07 Pat:      “Thiss (0.5) is nothing you can do
08           about i[t,
09 Doc:             [Okay.
10 Pat:      you just live with i::↑:t”
11           (0.6)
12 Doc: ->   But you think- so coming back to what
13      ->   you were saying befo:re for a second Lynn,
14      ->   part of it is the fear of (0.3) what might happen?
15 Pat:      .hhh I'm I'll be honest
16           [I've   ] never been frightened of dying,
17 Doc:      [(Yeah.)]
18 Doc:      No.
19 Pat:      .hhh until juss lately.
20 Doc:      Right.
```

Extract 7. Hospice day therapy consultation with a male patient diagnosed with motor neurone disease.

```
VERDIS02 07,48 VT61 EL02.2 MP
Dr A
Time into the consultation: 6.30 minutes

01 Pat:      I d- cuz I d- I don't know whether it's worth carrying on
02           taking (any) pills, as well, and just sit here.
03           (0.2)
04 Doc:      Mm:.              {Doc nods}
05           (0.5)
06 Pat:      And um
07           (1.1)
08 Doc: ->   You mentioned about a feeling of (0.3)
09      ->   throwing in the ↑towel
10           (0.4)
11 Pat:      Yeah stop taking the pills
12           and stuff like that iss (0.2)
13 Doc:      S↑top ↓taking the pills.=
14           =[That's what you mean] by [that?
15 Pat:      =[Yea:::h             ]    [Yea:::::::::::[:h.
16 Doc:                                                [O↑kay
```

Extract 8. From the same consultation as Extract 7 – male patient diagnosed with motor neurone disease.

```
VERDIS02 24,44 VT234 EL02.3 MP
Dr A
Time into the consultation: 23 minutes

01 Doc:      Do you feel anxious about things Ian?
02           (0.6)
03 Pat:      Um (4.2) to tell you the truth al t- a- al ne↓ver (0.4)
04           tlk right, (0.2) (↑it) (er-) (1.0)
05           (it er hhh) (.) No I suppose al ne↓ver .hhh
06           not for a while [and um (3.4) I think (1.1)
07 Doc:                     [(Mm) {Doc nods}
08 Pat:      when Bill died hhhh,
09 Doc:      ↑Mm  {Doc nods}
10           (1.0)
11 Pat:      I think tha- put- brought everything
12           back home you know?
13           (.)
14 Doc:      ↑Mm  {Doc nods}
15           (0.7)
16 Pat:      Um
17           (2.9)
18 Pat:      As it seems so unexpected as we:::[ll.
19 Doc:                                        [↑M::m  {Doc nods}
20 Pat:      .ksHHH (0.3) tklk and that (1.6)
21           and=hh (2.2) hhhh (0.3) don't know.
                    {Pat: slight lateral head shake}
22 Doc: ->   Did it make you th- think about you. hh
23 Pat:      YEA::::h. [Yeah it did at the time,=
24 Doc:                [Mm
25 Pat:      = a- .hh before all that (0.4)
26           you know I thought we::ll you're going to die
27           one day (this and [that but)
28 Doc:                        [Mm mm.
29           (0.5) {Doc nods}
31 Pat:      You know (↑i) but- (1.0) but now um (1.9)
32           I thin- I do think more of it,
```

Extract 9. Hospice outpatient consultation with a male patient diagnosed with pulmonary fibrosis, and his wife.

```
VERDIS23 37,35 VT891 EL23.5 MP ((audio only))
Dr F
Companion: patient’s wife
Time into the consultation: 33.20 minutes

01 Com:      He mainly stays in the car (don’t you?)
02           (0.3)
03 ???:      (Okay,)
04           (1.0)
05 Doc:      Okay.
06           (0.3)
07 Doc:      .hhhhhhhhhhh (0.4) Um (.) you said you turn things
08           over a bit at night. But you’re not (0.2)
09           really thinking that that’s keeping you awake,
10 Pat:      Mm
11 Doc:      In the day do you (.) do you worry about things?
12      ->   Do you- you- you- you mentioned about the (0.2)
13      ->   the cyanosis and (.) and also the oxygen, .hhh (.)
14      ->   um (1.5) you’ve heard what (.) doctor Fairclough
15      ->   sai[d,
16 Pat:         [Mm.
17           (0.4)
18 Doc: ->   you’ve had that (0.4) um pirfenidone, and it didn’t (0.2)
19      ->   didn’t (.) do [much and you’re off it,
20 Pat:                    [Yeah.
21 Doc: ->   .hhhh (0.7) do you worry about (0.9) what’s coming?
22           (0.5)
23 Pat:      tk U:::m:: (1.0) n::=no not really.
24           (1.3)
25 Pat:      It’s jus- (0.8) how how far along the line
26           I’m going to go before (0.4) I get to that h
27           (0.2)
28 Com:      M[m
29 Doc:       [Okay.
30 Pat:      yeah breathing wise, am I (0.6)
31           you know (0.3) >gonna be at a point where< (0.6)
32           I’m (0.7) really really struggling,
```

Extract 10. From the same consultation as Extract 1 – male patient diagnosed with neck tumour and lung metastases, and his brother.

```
VERDIS31 EL31.3 27.16 VT482 MP line 135
Dr D
Companion: patient’s brother
Time into the consultation: 28 minutes

((The patient has been talking about a clinical trial in which he hopes to
enrol. He refers to this at line 2))

01 Pat:      I don’t know what they’re going to do.
02           .hhhh But they are gonna call me.
03           (0.4)
04 Doc:      O[kay.
05 Pat:       [only if they get everything there.
06 Doc: ->   Okay. So that’s one (ha-) that’s one ↓thing
07      ->   is hoping that the Royal will have
08      ->   trea[tment [that’ll work,]
09 Pat:          [Yeah.
10 Com:                 [(Yeah.)      ]
11 Doc: ->   .hh[hhhh do you ever wonder what will happen=
12 Com:         [Yeah.
13 Doc: ->   =if they don’t have treatment that works?
14 Pat:      (hho) No::.
15 Doc:      ‘Cause you [said
16 Pat:                 [(It’s a long waiting there,)
17 Doc:      ↓Yeah
18 Pat:      There is nothing ↑else
19           (.)
20 Doc:      No.
21 Pat:      .hhh [They  have been very] honest. =
22 Doc:           [(An’) you said some-]
23 Pat:      = There’s nothing ↑else
24 Doc:      No.
25 Pat:      .hh because eh (t-t-ay) you guys
26           gave me a lot of (0.3) .hhh chemo:,
27           and there is nothing e- (.) mo:re [chemo =
28 Doc:                                        [No.
29 Pat:      = you guys can give me?
30 Doc:      [No.
31 Pat:      [.hhhh Radiotherapy:
32           last time he said “I won’t give you anymore”,
33           but he .hhh luckily gave me one mo:re, (.)
34 Doc:      ↑Mm
35 Pat:      just to (0.3)
36 Doc:      ↑Mm
37           (0.3)
38 Pat:      Yeah it’s a long wait now,
39           (a)hhh I’ve [got  no  i]dea what
40 Doc:                  [(It’s a long wait)]
41 Doc:      Mm [↑mm
42 Pat:         [.hh and one thing is (0.6)
43           I’m getting worse.
44 Doc:      Ye:s.
45           (0.3)
46 Pat:      So: my mind plays up a little bit,
47 Doc:      [Mh.
48 Pat:      [(          ) I hope it’s not (.)
49           my time y↑et hah.
```

```
50 Doc:     Y↑eah
51          (0.2)
52 Pat:     Be[ing very honest,
53 Doc:       [‘Cause you-
54 Doc:     ‘Cause you said when the coughing was bad,
55           sometimes you wonder [if it might be then?]
56 Pat:                           [Oh yeah.  (It comes)]
57 Pat:     (You know that whe) .hhh When the coughing
58          is really bad, it’s [just (0.7)
59 Doc:                        [Mh,
60 Pat:     hh I think about i(t) (a)hh
61          .hhh[hh sometimes you don’t wanna think about it
62 Doc:         [Mm.
63 Pat:     but it just ↑comes
```
