# Supplementary material for: Engaging Terminally Ill Patients in End of Life Talk: How Experienced Palliative Medicine Doctors Navigate the Dilemma of Promoting Discussions about Dying
Source: PLoS One. 2016 May 31;11(5):e0156174. doi: 10.1371/journal.pone.0156174 (PMC4887020; doi:10.1371/journal.pone.0156174)
Supplement: S2 File — (PDF) [file pone.0156174.s002.pdf]

Supporting information to Pino M, Parry R, Land V, Faull C, Feathers L, and Seymour J (2016). Engaging terminally ill patients in end of life talk: How experienced palliative medicine doctors navigate the dilemma of promoting discussions about dying. *PLOS ONE*.

S2 File. Transcription conventions; simplified version based on Gail Jefferson's work [44].

Doc, Pat, Com	Participant role (Doctor, Patient, Companion)
[	Left-side brackets indicate where overlapping talk begins.
]	Right-side brackets indicate where overlapping talk ends.
=	Equal signs (ordinarily at the end of one line and the start of an ensuing one) indicate a 'latched' relationship – no silence at all between them.
(0.8)<br>(.)	Numbers in parentheses indicate silences in tenths of a second. A period inside parentheses is a silence less than two-tenths of a second.
wo:::rd	Colons indicate a lengthening of the sound just preceding them, proportional to the number of colons.
wo-	A hyphen indicates an abrupt cut-off or self-interruption of the sound in progress indicated by the preceding letter.
, ? .	Punctuation captures intonation, not grammar: *comma* is for slightly upward 'continuing' intonation; *question mark* for marked upward intonation; and *period* for falling intonation.
word	Underlining indicates stress or emphasis (usually conveyed through slightly rising intonation).
wo:rd<br>wo:rd	Combinations of underlining and colons indicate intonation contours. An underscored letter followed by colon indicates a falling intonation contour. A letter followed by an underlined colon represents a rising intonation contour.
↑ ↓	An arrow symbol indicates a marked pitch rise or fall of the sound or syllable after the arrow.
( )<br>(word)	Empty parentheses indicate inaudible talk. Words or letters inside such parentheses indicate a best estimate of what is being said.
hhh<br>.hhh	The letter ''h'' is used to indicate hearable aspiration, its length roughly proportional to the number of h's. If preceded by a dot, the aspiration is an in-breath.
w(h)ord	Aspiration internal to a word is represented through an "h" enclosed in parentheses (h), such as when people produce particles of laughter throughout the production of a word.
°word°<br>°°word°°	Talk bounded by degree symbols is quieter than the surrounding talk. Talk bounded by double degree symbols is whispered.
WORD	Uppercase indicates talk that is louder than the surrounding talk.
{Words}	Words in braces describe participants' non-verbal actions (e.g. gestures).
((words))	Words in double parentheses indicate transcriptionist's comments.
->	Arrows are used to highlight doctors' actions that are the focus of the analysis – i.e. their elaboration solicitations.
